# Nutrient Removal Potential of Headwater Wetlands in Coastal Plains of Alabama, USA

**DOI:** 10.3390/w15152687

**Published:** 2023-07-25

**Authors:** Sabahattin Isik, Henrique Haas, Latif Kalin, Mohamed M. Hantush, Christopher Nietch

**Affiliations:** 1 College of Forestry, Wildlife and Environment, Auburn University, 602 Duncan Drive, Auburn, AL 36849, USA; 2 U.S. EPA Center for Environmental Solutions and Emergency Response, 26 West Martin Luther King Dr., Cincinnati, OH 45268, USA; 3 U.S. EPA Center for Environmental Measurement and Modeling, 26 West Martin Luther King Dr., Cincinnati, OH 45268, USA

**Keywords:** watershed modeling, wetland modeling, water quantity/quality prediction, SWAT, WetQual, uncertainty, Fish River

## Abstract

Headwater streams drain over 70% of the land in the United States with headwater wetlands covering 6.59 million hectares. These ecosystems are important landscape features in the southeast United States, with underlying effects on ecosystem health, water yield, nutrient cycling, biodiversity, and water quality. However, little is known about the relationship between headwater wetlands’ nutrient function (i.e., nutrient load removal $\left(R_{L}\right)$ and removal efficiency $\left(E_{R}\right)$) and their physical characteristics. Here, we investigate this relationship for 44 headwater wetlands located within the Upper Fish River watershed (UFRW) in coastal Alabama. To accomplish this objective, we apply the process-based watershed model SWAT (Soil and Water Assessment Tool) to generate flow and nutrient loadings to each study wetland and subsequently quantify the wetland-level nutrient removal efficiencies using the process-based wetland model WetQual. Results show that the calculated removal efficiencies of the headwater wetlands in the UFRW are 75–84% and 27–35% for nitrate $\left({\text{NO}}_{3}^{{}^{-}}\right)$ and phosphate $\left({\text{PO}}_{4}^{+}\right)$, respectively. The calculated nutrient load removals are highly correlated with the input loads, and the estimated ${\text{PO}}_{4}^{+}\;E_{R}$ shows a significant decreasing trend with increased input loadings. The relationship between ${\text{NO}}_{3}^{{}^{-}}\;E_{R}$ and wetland physical characteristics such as area, volume, and residence time is statistically insignificant (*p* > 0.05), while for ${\text{PO}}_{4}^{+}$, the correlation is positive and statistically significant (*p* < 0.05). On the other hand, flashiness (flow pulsing) and baseflow index (fraction of inflow that is coming from baseflow) have a strong effect on ${\text{NO}}_{3}^{{}^{-}}$ removal but not on ${\text{PO}}_{4}^{+}$ removal. Modeling results and statistical analysis point toward denitrification and plant uptake as major ${\text{NO}}_{3}^{{}^{-}}$ removal mechanisms, whereas plant uptake, diffusion, and settling of sediment-bound P were the main mechanisms for ${\text{PO}}_{4}^{+}$ removal. Additionally, the computed nutrient $E_{R}$ is higher during the driest year of the simulated period compared to during the wettest year. Our findings are in line with global-level studies and offer new insights into wetland physical characteristics affecting nutrient removal efficiency and the importance of headwater wetlands in mitigating water quality deterioration in coastal areas. The regression relationships for ${\text{NO}}_{3}^{{}^{-}}$ and ${\text{PO}}_{4}^{+}$ load removals in the selected 44 wetlands are then used to extrapolate nutrient load removals to 348 unmodeled non-riverine and non-riparian wetlands in the UFRW (41% of UFRW drains to them). Results show that these wetlands remove 51–61% of the ${\text{NO}}_{3}^{{}^{-}}$ and 5–10% of the ${\text{PO}}_{4}^{+}$ loading they receive from their respective drainage areas. Due to geographical proximity and physiographic similarity, these results can be scaled up to the coastal plains of Alabama and Northwest Florida.

## 1. Introduction

Wetlands can be considered as one of the most efficient best management practices (BMPs) for pollutant removal [1]. These ecosystems are recognized for their significant ecological and economic values and the wide variety of ecosystem services they provide, such as water quality purification, material transformation, carbon sequestration, flood control, wildlife habitats, and the biodiversity they offer at the ecosystem and watershed level [2]. Natural and constructed wetlands can reduce peak flows and runoff volume to a certain degree, in addition to providing considerable esthetic and wildlife benefits. Wetlands can remove excess inorganic and organic nutrients from sources such as fertilizer runnoff and failing septic systems. They can filter sediments washed off during soil erosion and trap pollutants such as pesticides and some heavy metals. These materials can seriously degrade the quality of surface water and groundwater resources. Wetlands naturally retain and “recycle” nutrients, delaying downstream export. Wetlands are critical watershed features in many systems, regulating local and regional hydrology and the fate and transport of various water quality constituents [3].

Although natural wetlands can play a vital role in hydrological regimes and biogeochemical processes [4], they have not received a level of attention in nutrient retention studies comparable to other lentic systems such as lakes and reservoirs [5–8]. While processes controlling nutrient retention in aquatic ecosystems are relatively well understood [9,10], for natural wetlands, information related to nutrient removal rates among multiple systems dispersed over a local catchment or regional watershed is currently lacking. Global-scale studies have considered wetlands’ efficiency in removing nutrient loads. For instance, Jordan et al. [11] conducted a meta-analysis of 190 wetland systems worldwide and found a strong and positive relationship between total reactive nitrogen (TNr) removal and reactive nitrogen loadings. The authors also estimated that, worldwide, approximately 17% of the total anthropogenic load of TNr to aquatic ecosystems is removed by wetlands. Similarly, Cheng and Basu [9] analyzed data from 600 lentic systems across the globe, including wetlands, lakes, and reservoirs, and found that smaller wetlands (<316 m^2^ in surface area) are responsible for 50% of the total nitrogen (TN) removal. Studies such as those by Fisher and Acreman [12] and Land et al. [13], who reviewed data from 57 and 207 wetlands, respectively, also showed the importance of these systems in removing N and phosphorus (P) loadings. Fisher and Acreman [12] reported 80% and 84% of N and P removal, respectively, for the 57 wetlands they examined, but some wetlands increased soluble N and P loading. Land et al. [13] reported median removal efficiencies of N and P of 37% (29–44%, 95% confidence interval (CI)) and 46% (37–55%, CI), respectively. Under moderate-to-high streamflow conditions, Hansen et al. [14] found wetlands in the Minnesota River basin to be five times more efficient (per unit area basis) at reducing riverine nitrate concentration than highly effective land-based nitrogen mitigation strategies such as cover crops and land retirement. However, such studies did not examine specific physical and hydrologic characteristics of the systems contributing to the effectiveness or provide nutrient source–sink relationships at local catchment or regional scales.

Monitored flow and nutrient data at wetland inlets and outlets are rarely available, and setting up gauging stations at desired locations to collect data for a sufficiently long period of time can be impractical [15]. As a result, field studies are usually restricted to monitoring one or only a few wetlands, as in [16–18], and often do not provide sufficient information on the relationship between nutrient removal rates and wetland characteristics (geomorphologic, hydraulic, and hydrologic) and nutrient loads. Alternatively, models have been increasingly used in data-sparse watersheds to study wetland ecosystems and their effects on watershed-scale water quantity and quality [9,19–23]. However, except for those of Czuba et al. [22] and Cheng and Basu [9], most of these studies relied on empirical models or models that oversimplify the biogeochemical processing occurring within wetlands. For instance, Uuemaa et al. [21] applied a simple mass-balance-based model relying on hourly measurements of wetland inflow, outflow, rainfall, and Penman–Monteith evapotranspiration estimates to assess the removal of N in a single wetland in New Zealand. Liu et al. [20] introduced a GIS-based distributed wetland modeling system and assessed the aggregate effect of 492 wetlands on the watershed’s sediment, total N, and total P yields in a small watershed in Canada. The model uses a very simplistic mass balance approach for wetland sediment and nutrient routing borrowed from the wetland water quality algorithm of the Soil and Water Assessment Tool (SWAT) model.

Although valuable, studies relying on empirical or simplistic wetland models are usually of limited utility and cannot be extrapolated outside the range of the empirical measurements, nor can they be generalized to represent hydrological and biogeochemical processes in natural wetlands with a high degree of fidelity. As highlighted by Ghermandi et al. [24], all wetlands are not equal, and thus studies aimed at understanding the wetland physical characteristics affecting the removal of nutrient loadings must rely on adequate tools. This is particularly important for planning watershed or landscape-level restoration efforts, since an understanding of how different types of wetlands respond to nutrient processing and pollutant removal may help to protect these ecosystems and minimize excess nutrients loadings to surface water bodies downstream [9,10]. Progress in modeling restored and engineered wetlands has outpaced that for natural wetlands. Much less progress has been made in process-based modeling of natural inland and coastal wetlands impacted by variably saturated soils or tidal effects (e.g., [25,26]). For instance, Sharifi et al. [26] developed a process-based model capable of simulating nutrient cycling and biogeochemical processes in ponded and unsaturated wetland soils.

Our research goal is to estimate the cumulative effects on nutrient reduction of multiple natural wetlands dispersed over larger watershed scales. To the best of the authors’ knowledge, systematic process-based modeling studies on watershed-scale cumulative wetland nutrients reduction, especially in sparsely monitored or ungauged basins, are rare research endeavors. In this study, we use an integrated modeling framework to explore the relationship between nutrient removal and nutrient loading as a function of wetland physical characteristics (area, volume, and residence time) and hydrologic characteristics (flashiness and baseflow indices) in 44 wetlands located at the headwaters of the Upper Fish River watershed (UFRW) in coastal Alabama. To better understand the factors impacting the removal efficiencies in the headwater wetlands, we apply a process-based watershed model, namely, SWAT [27], to generate flow and nutrient loadings to the wetlands and subsequently quantify the wetland-level nutrient removal efficiencies using the process-based wetland model WetQual [28–30]. The UFRW drains to Weeks Bay, a sub-estuary bordering Mobile Bay, Alabama, USA [31,32].

Coastal areas such as the Mobile Bay region are expected to witness higher population growth compared to non-coastal areas [33,34]; consequently, excess nutrient discharges to surface waters are expected. In addition, the accompanying increased urbanization activities such as channel modification, water diversion, and land development threaten headwater wetlands by increasing erosion, sedimentation, and desiccation [35–37]. Therefore, it is particularly important for water quality protection in coastal areas to assess the importance of headwater wetlands and the factors governing the removal or sequestration of nutrients. Some studies have shown that headwater wetlands have a greater effect in mitigating nonpoint source pollution compared to more downstream wetlands such as floodplain wetlands [38,39]. Therefore, protecting these ecosystems appears to be vital for sustainable watershed development.

Our specific research objectives are to: (i) quantify nutrient input loadings to headwater wetlands in a watershed with sparse wetland nutrient data; (ii) demonstrate the utility of a process-based model framework to fill in the data gap; (iii) quantify uncertainties associated with the estimated reductions and removal efficiencies; (iv) use model outputs to develop regression relationships between nutrient loads and removals and use the relationships to estimate nutrient removals for similar headwater wetlands; (v) explore correlations between nutrient removal efficiency and wetland characteristics; and (vi) scale up headwater wetland nutrients reductions from the UFRW to the coastal plains of Alabama and Northwest Florida.

## 2. Study Area

**Figure 1.**
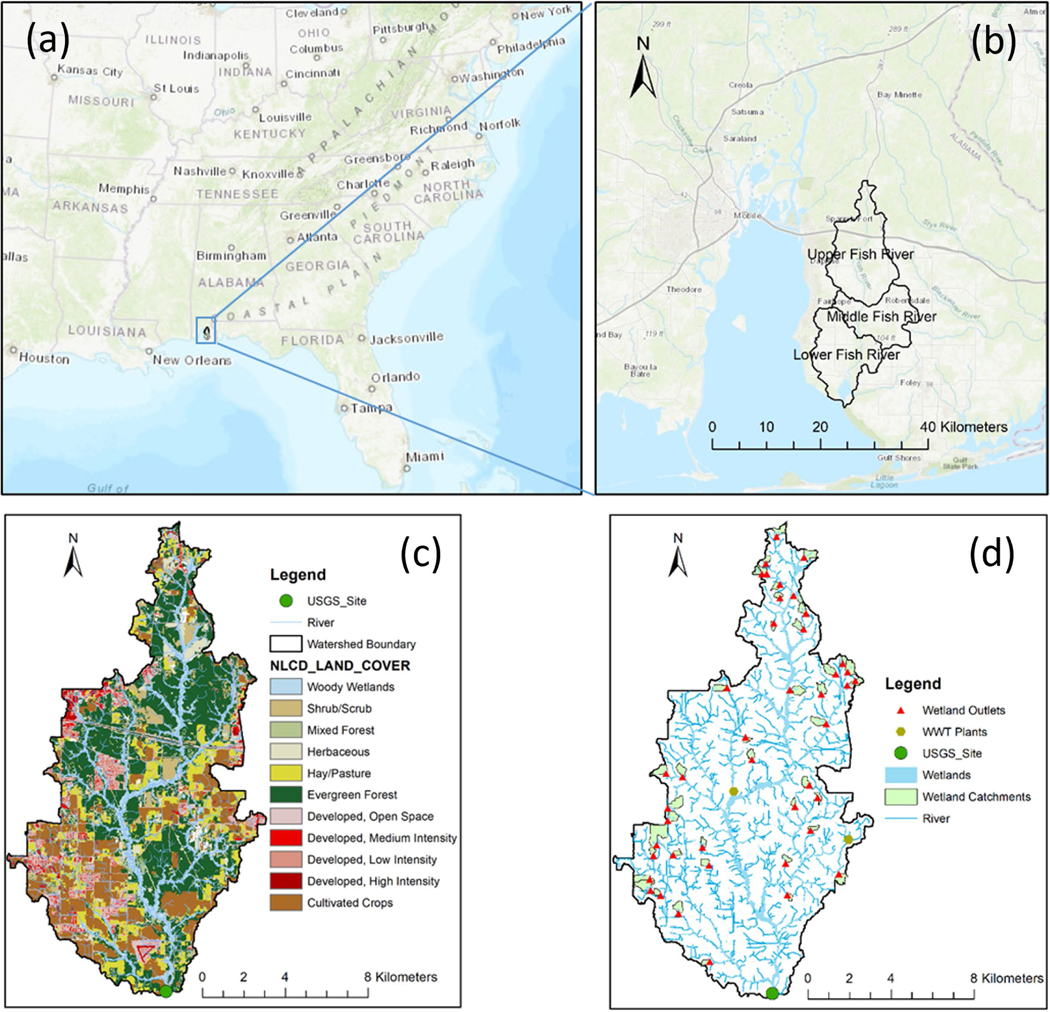
Location of the Fish River watershed in coastal Alabama (**a**), HUC12 boundaries of the Fish River watershed (**b**), land use/cover of the UFRW (**c**), and spatial distribution of the selected wetlands along with their drainage catchments in the UFRW (**d**).

The Fish River watershed, located in southern Baldwin County in coastal Alabama (Figure 1a), drains approximately 73% of the total freshwater flowing into Weeks Bay, which is part of the Mobile Bay estuarine system along the northern Gulf of Mexico [31,32]. The watershed has an area of approximately 408.6 km^2^ and is comprised of the following three USGS 12-digit Hydrologic Unit Code (HUC) areas: Upper Fish River (HUC 031602050201), Middle Fish River (HUC 031602050202), and Lower Fish River (HUC 031602050204) (Figure 1b). This study focused on the UFRW. The UFRW was selected because of the available long-term records of streamflow and water quality (e.g., nitrate and phosphate) data collected at the watershed’s outlet, the abundance of headwater wetlands, and the increasing urbanization rates witnessed in the last few decades [40–42].

The UFRW study area is in a humid subtropical region of the U.S. Gulf Coast. Summers are typically warm and humid, with winters being mild with occasional cold spells. The average annual precipitation of the region is 1680 mm. The average annual air temperature is approximately 20 °C, with an average January temperature of 10.5 °C and an average July temperature of 28 °C. According to NCLD 2016 [43], the watershed’s LULC mainly consists of forest (33%), agriculture (18%), urban (14%), hay/pasture (12%), and forested wetlands (11%) (Figure 1c). Wetland data were obtained from the U.S. Department of the Interior’s Fish and Wildlife Service National Wetland Inventory (NWI) (http://www.fws.gov/wetlands, accessed on 15 April 2021). According to the NWI database, there is a total of 1153 wetlands, which includes freshwater forested/shrub wetlands, freshwater emergent wetlands, riparian wetlands, and riverine wetlands. The total area of these wetlands is 1181 ha across the UFRW. Out of these, 754 are freshwater ponds, freshwater forested/shrub wetlands, and emergent wetlands, occupying an area of 663 ha, which are mostly headwater wetlands and are the focus of this study. The riparian and riverine wetlands and lakes were not considered.

**Figure 2.**
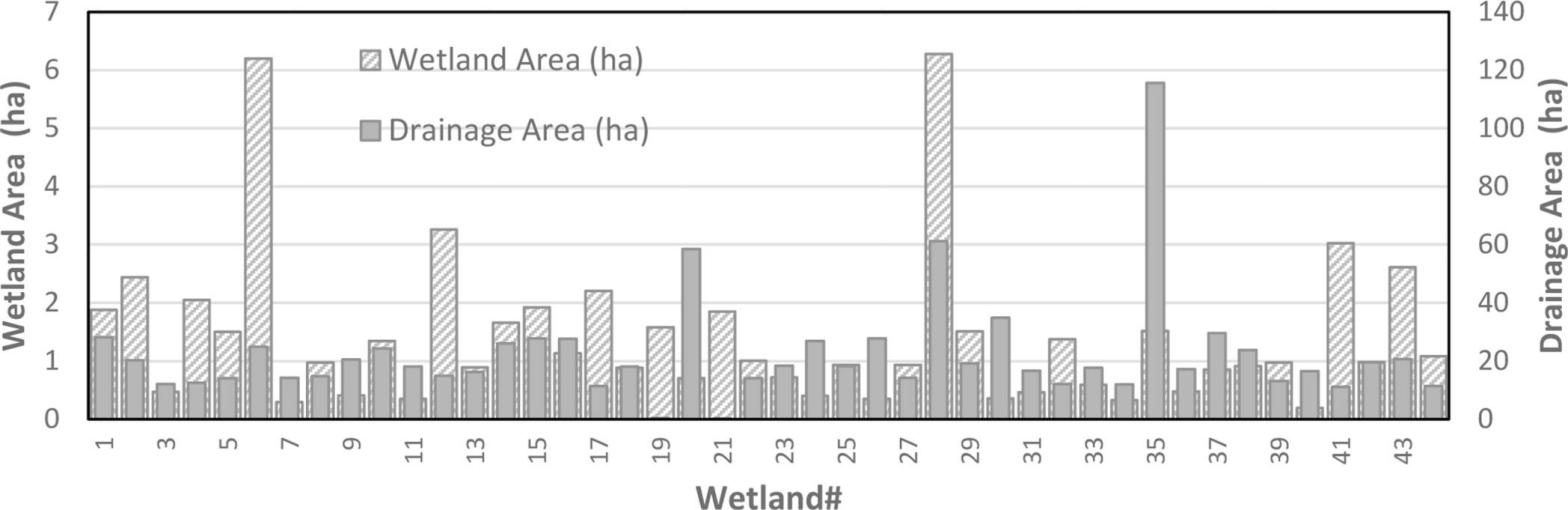
Surface areas of the wetlands in the UFRW (based on U.S. Wetland Inventory) and their watershed areas.

It is worth mentioning that the total surface area of these forested and emergent freshwater wetlands covers approximately 4.6% of the whole watershed area (14,382 ha), making them a significant physiogeographic feature of the UFRW. Figure 1d shows the 44 headwater wetlands analyzed in this study, comprised mostly of forested wetlands. Wetlands were identified using the data layer downloaded from the NWI Inventory website (https://www.fws.gov/wetlands, accessed on 15 April 2021). Using ArcGIS (ESRI), the downloaded data were intersected with the UFRW boundary, and the drainage area for each selected wetland was delineated. Following Colvin et al. [37], we considered wetlands on the first- and second-order streams as headwater wetlands. After assigning stream order to the National Hydrography Dataset (NHD) stream network, a buffer of 250 m was created around first- and second-order streams. All wetlands within this buffer area were selected as headwater wetlands. These selected wetlands were cross-checked for accuracy using background aerial imagery to eliminate potential errors associated with the National Wetland Inventory data. After inspection of the selected wetlands, 44 headwater wetlands significant in size, spread out over the watershed, were selected for analysis. The total surface area of the selected wetlands is approximately 62 ha. Figure 1d shows the identified wetlands as coordinate points and their respective upstream drainage areas, and Figure 2 plots each wetland’s area, as well as its catchment area, which varies from 0.35 to 115.6 ha (total 977.6 ha).

## 3. Methods

### 3.1. Modeling Framework

The process-based WetQual model [26,28–30] was used to evaluate the pollutant removal efficiencies of the 44 selected wetlands. Due to the lack of observed discharge and loadings draining to each of these wetlands, the watershed-scale hydrologic model SWAT [27] was used to model terrestrial generation and transport of flow, ${\text{NO}}_{3}^{{}^{-}}$, and phosphate $\left({\text{PO}}_{4}^{+}\right)$. Studies have shown that natural wetlands are more effective in removing inorganic forms of nutrients compared to organic forms [9]. Further, we had ${\text{NO}}_{3}^{{}^{-}}$ and ${\text{PO}}_{4}^{+}$ data at a USGS gauge at the outlet of the UFRW to calibrate SWAT. Although ammonia data were available at the same site, the concentrations were extremely low; thus, ammonia removal was not considered in this study. SWAT is widely used to assess the impacts of anthropogenic activities on watershed-scale water quantity [44–47] and quality [48–51]. As a semi-distributed model, SWAT divides the watershed into sub-basins, which are further discretized into unique combinations of land use, soil, and slope known as hydrologic response units (HRUs). A SWAT model comprised of 1549 sub-basins and 15,519 HRUs was built to accurately capture the selected wetlands and the upstream areas draining to them. To accomplish this, a 10-meter resolution DEM (Digital Elevation Model) and stream network shapefile derived from the National Hydrography Dataset Plus (NHDPlus) were used to delineate the drainage areas to each wetland. The selected wetlands were georeferenced to generate flow and nutrient loadings through SWAT to each wetland. Under this rationale, SWAT acts as the loading model whilst WetQual serves as the receiving model. Next, WetQual was run for each wetland with the SWAT-generated loadings to simulate the flow and nutrient loads leaving the wetlands, especially ${\text{NO}}_{3}^{{}^{-}}$ and ${\text{PO}}_{4}^{+}$.

The WetQual model requires daily runoff and nutrient loadings as input. Reach-level daily streamflow estimates from SWAT were used as inflows into the wetland systems. Wetland outflows were estimated using a simple continuity equation to perform flow routing in a wetland system [28]:

(1) $$\phi_{w}\frac{dV_{w}}{dt}=Q_{i}+Q_{g}-Q_{o}-AE_{T}+Ai_{p}$$

where $\phi_{w}$ is the effective porosity (due to the presence of vegetation) of wetland surface water; $V_{w}$ is the volume of standing water in the wetland $\left[{\text{L}}^{3}\right]$; *t* is time [T]; $Q_{i}$ is the volumetric inflow rate $\left[{\text{L}}^{3}{\text{T}}^{-1}\right]$; $Q_{g}$ is the groundwater discharge (negative for infiltration) $\left[{\text{L}}^{3}{\text{T}}^{-1}\right]$; $Q_{o}$ is the wetland discharge rate $\left[{\text{L}}^{3}{\text{T}}^{-1}\right]$; A is the wetland surface area $\left[{\text{L}}^{2}\right]$; $E_{T}$ is the evapotranspiration rate $\left[{\text{L}}^{3}{\text{T}}^{-1}{\text{L}}^{-2}\right]$; and $i_{p}$ is the precipitation rate $\left[{\text{LT}}^{-1}\right]$. $V_{w}$ and $Q_{o}$ were calculated using the elevation–area–volume relationships of the selected 44 wetlands in the UFRW, which were determined using the ArcGIS Spatial Analyst Supplemental Tools (ESRI, 2015). Since these are natural wetlands and have no outlet structures to provide depth/volume vs. outflow relationships, wetland outflows were estimated using the following equations, which were adapted from SWAT [52]:

(2) $$\begin{array}{cc} V_{o}=0 & \mathrm{if}V_{w}<V_{n}\\ V_{o}=\frac{V_{w}-V_{n}}{c} & \mathrm{if}V_{n}\leq V_{w}\leq V_{m}\\ V_{o}={\left(V_{w}-V_{m}\right)}^{2}+\frac{V_{m}-V_{\mathrm{nor}}}{c} & \mathrm{if}V_{w}>V_{m}\left(V_{n}=V_{m}/p\right) \end{array}$$

where $V_{o}$ is the volume of water flowing out of the wetland during the day (L^3^), $V_{w}$ is the volume of water stored in the wetland $\left({\text{L}}^{3}\right)$, and $V_{n}$ and $V_{m}$ are the volume of water held in the wetland when filled to the normal and maximum water level $\left({\text{L}}^{3}\right)$, respectively. Outflow from the wetland starts at $V_{n}$ level. The water volume of the wetland reaches its maximum capacity at $V_{m}$ level, which was found using ArcGIS Spatial Analyst Supplemental Tools [53] at the maximum wetland area in the NWI database. The parameters *c* and *p* are constants. We examined the sensitivity of *c* and *p* parameters using monitored flow and bathymetry data at three headwater wetlands which are very close to the UFRW in Baldwin County, Alabama [18]. The values of *c* and *p* varied from 1 to 20 and from 1 to 10, respectively. The maximum difference in *R*^2^ between observed and simulated outflows at the three sites was 1.5% when the *c* and *p* were varied within those ranges, meaning model flow estimates were not very sensitive to *c* and *p*. Hence, we assumed that the normal water level is equal to a tenth of the maximum depth (*p* = 10), and *c* = 10, as suggested in the SWAT Theoretical Documentation [52].

In addition to discharge, nutrient, and sediment loadings, climate data such as precipitation, air temperature, and potential evapotranspiration values were also transferred from SWAT to WetQual. The SWAT and WetQual models were run for each headwater wetland and contributing drainage area for the period 2004–2015, with the initial four years used as a warm-up period. Therefore, all the analyses were carried out for the 2008–2015 period. For further model details, including SWAT and WetQual setup, model inputs, and calibration, the reader is referred to the Supplementary Materials section (Sections S1–S3).

### 3.2. Model Uncertainty

**Figure 3.**
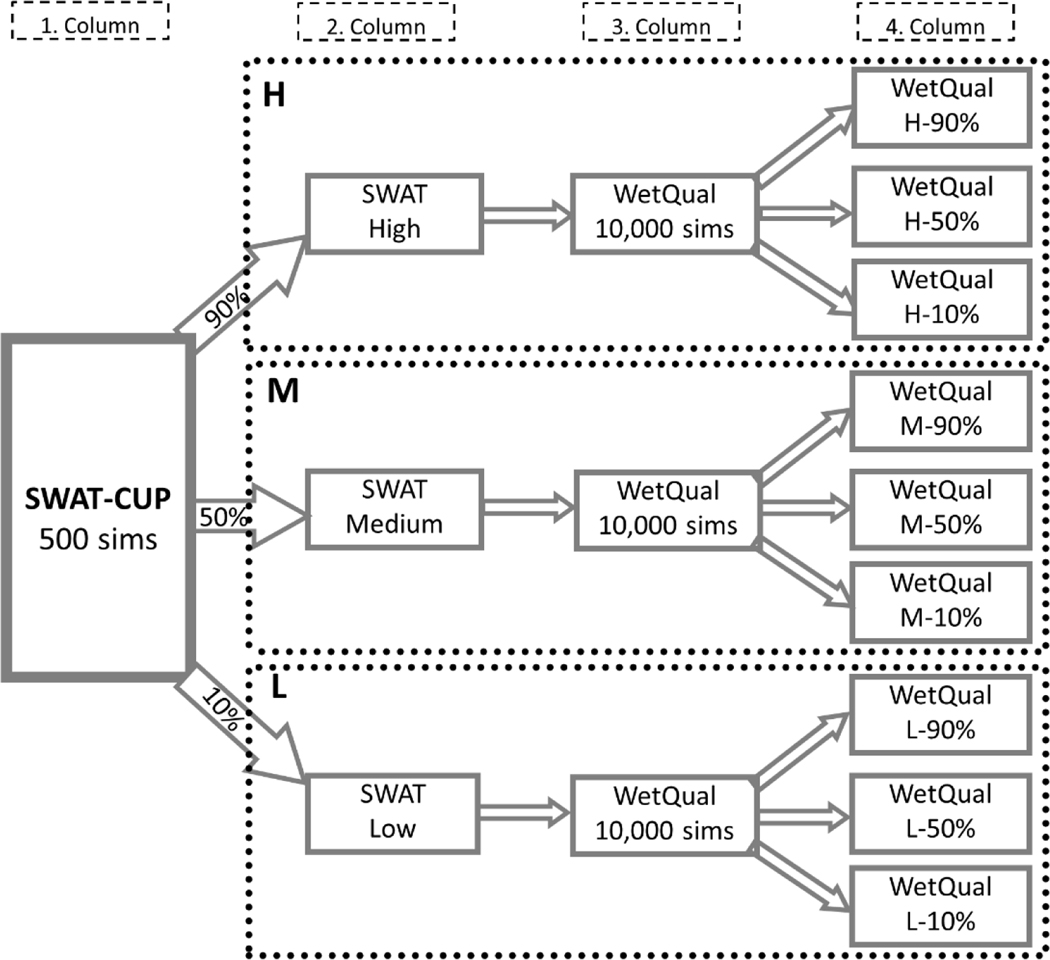
Integration of SWAT and WetQual with consideration of uncertainty. Please see sections “Modeling Framework” and “Model Uncertainty” for details. Low, Medium, and High in column 2 are nutrient loads at wetland inlets at the 10th, 50th, and 90th percentile of SWAT-CUP simulations, respectively. X-10%, X-50%, and X-90% are, respectively, the 10th, 50th, and 90th percentiles of WetQual simulations at wetland outlets corresponding to the low, medium, or high loads (X).

Estimating model predictive uncertainty provides environmental managers a basis for selecting among alternative actions and assessing how different model outputs affect the response of the studied system [29,54]. Figure 3 is a flowchart schematic of our modeling framework and how uncertainty was addressed. All the processes in Figure 3 were separately implemented for ${\text{NO}}_{3}^{{}^{-}}$ and ${\text{PO}}_{4}^{+}$.

We carried out a Monte-Carlo-based uncertainty analysis (MCUA) to address the uncertainty of the SWAT and WetQual simulated loadings. MCUA is widely used to evaluate the uncertainty associated with numerical models and is based on the statistical analyses of the outputs of multiple model runs [55]. The following steps were implemented for estimating uncertainty in the SWAT and WetQual outputs:

(i) The SWAT model built for the UFRW was stochastically calibrated for monthly streamflow, ${\text{NO}}_{3}^{{}^{-}}$, and ${\text{PO}}_{4}^{+}$ at the watershed outlet using the SWAT Calibration and Uncertainty Program (SWAT-CUP) [56]. We performed several iterations of Monte Carlo (MC) simulations, with each iteration consisting of 500 simulations. Each simulation corresponded to a parameter set randomly generated by SWAT-CUP. The final iteration, which had the best model performance based on Nash–Sutcliffe efficiency and the smallest 95% confidence interval, was selected as the calibrated model (column 1 in Figure 3). We ordered the 500 parameter sets from the final iteration according to the percent bias (*PBIAS*) objective function to identify low, medium, and high loadings, which were considered to represent the 10th, 50th (median), and 90th percentile loadings, respectively;
(ii) SWAT was run at a daily time-step using the parameter sets identified instep (i) (column 2 in Figure 3) to generate the low, medium, and high loadings of ${\text{NO}}_{3}^{{}^{-}}$ and ${\text{PO}}_{4}^{+}$ to each wetland;
(iii) Daily SWAT outputs were fed as input to the WetQual model to perform MCsimulation (10,000 model runs) for each of the low, medium, and high loading timeseries for ${\text{NO}}_{3}^{{}^{-}}$ and ${\text{PO}}_{4}^{+}$ (column 3 in Figure 3). This was conducted for each of the 44 headwater wetlands. The MC simulation produced 10,000 timeseries for each of the ${\text{NO}}_{3}^{{}^{-}}$ and ${\text{PO}}_{4}^{+}$ loads out of each of the 44 wetlands. This was accomplished by random sampling of 10,000 independent WetQual parameter sets from the calibrated (posterior) parameter distributions shown in Table S3. The MCUA-based calibration was achieved using observed data at a headwater wetland very close to the UFRW in Baldwin County, Alabama [18]. For more details on WetQual calibration using MCUA, please refer to Supplementary Materials;
(iv) The 10th, 50th (median), and 90th percentiles of average annual nutrient loads (out of the wetlands) were obtained from the MC simulation in step (iii) for low, medium, and high loadings computed by SWAT (column 4 in Figure 3). However, for brevity, only median annual loads are presented and discussed, with the results on the lower and upper percentiles summarized in the Supplementary Materials (Section S7).

It is worth mentioning that, although a full MCUA is desirable, it would be computationally too demanding. A full MCUA would require 500 (SWAT) × 10,000 (WetQual) × 44 (wetlands) × 2 (constituents) = 440 million model runs. Since this was not computationally feasible given our resources, we considered the 10th, 50th, and 90th percentiles of the MC simulation (10,000 runs) as a proper measure of WetQual uncertainty. As mentioned before, here we present the results based on the medians of WetQual simulated average annual loads corresponding to SWAT-computed low, medium, and high loadings.

A FORTRAN script was written to automate the multiple processes of reading, computing, and writing from SWAT-CUP outputs to compatible WetQual inputs. The script converts nutrient loads from SWAT’s reach-level output file to concentrations to be used as inputs in WetQual. The script also runs the wetland flow routing subroutine and WetQual model simulations and, finally, calculates the percentiles of WetQual outputs.

### 3.3. Assessment of Nutrient Removal Rates by Wetlands

We assessed the effectiveness of wetlands in nutrient removal using both load removal and relative removal rates. We calculated the average annual loads for each WetQual MC simulation for the period 2008–2015 and then used the median of the average annual loads for further analysis. For brevity, henceforth, we drop the word median from average annual load. This was repeated for each of the low, medium, and high loading timeseries obtained from SWAT. The calculated nutrient loads in or out of each wetland were scaled by dividing by the wetland area (kg/ha/y) using the wetland areas reported in NWI. Henceforth, we refer to nutrient loading as L for brevity, consistent with other studies on wetland nutrient removal [11,12]. Nutrient load removal per wetland area $\left(R_{L}\right)\left(\text{kg}/\text{ha}/\text{y}\right)$ was calculated by subtracting the nutrient load leaving the wetland $\left(L_{out}\right)$ from the nutrient load into the wetland $\left(L_{in}\right)$:

(3) $$R_{L}=\left(L_{\mathrm{in}}-L_{\mathrm{out}}\right)(\text{kg}/\text{ha}/\text{y})$$

Nutrient load removal efficiency $\left(E_{R}\right)$ was calculated as:

(4) $$E_{R}(\%)=100\frac{R_{L}}{L_{in}}$$

$R_{L}$ and $E_{R}$ at wetlands were plotted against $L_{in}$ to explore the effect of input loading on nutrient removal and removal efficiency. The relationship constructed between $L_{in}$ and $R_{L}$ could be useful in estimating nutrient removal at other headwater wetlands in the region. Henceforth, unless otherwise specifically indicated that they are calculated from observations, the phrases “load removal” and “removal efficiency”, respectively, refer to $R_{L}$ and $E_{R}$ computed by the SWAT-WetQual model framework. We plotted the nutrient removal efficiencies $\left(E_{R}\right)$ against wetland characteristics, such as wetland volume, the ratio of wetland area to the contributing drainage area, and residence time, to gain insights into the impact of these characteristics on $E_{R}$. The residence time (τ) was estimated by dividing the annual average wetland volumes by the period-averaged annual outflows.

The effects of flashiness and baseflow on nutrient reduction rates were investigated using the Richards–Baker flashiness index (*RBI*) [57] and the baseflow index (*BFI*). The *BFI* is the ratio of baseflow volume to streamflow volume and is an indicator for groundwater contribution to streamflow. The online digital filter developed by Lim et al. [58] was used to separate baseflow from streamflow. The *RBI* measures fluctuations in flow and can be computed as:

(5) $$RBI=\frac{\sum_{i=1}^{n}\left|Q_{i}-Q_{i-1}\right|}{\sum_{i=1}^{n}Q_{i}}$$

where $Q_{i}$ is the wetland outflow flow rate at the *i*^th^ day, and *n* is the total number of daily flows. *RBI* can also be considered as a metric-capturing flow pulsing at high temporal resolution (daily in this case), something similar to the flood pulsing concept [59,60].

Additionally, we compared nutrient removal efficiencies for dry and wet years. Temperature and evapotranspiration values were not significantly different among the years. The average annual precipitation was 1718 mm between 2008 and 2015, while the minimum and maximum precipitations were 1258 and 2050 mm in 2011 and 2014, respectively. Thus, we considered 2011 and 2014 as dry and wet years, respectively.

## 4. Results

### 4.1 . Nutrient Removals and Their Efficiencies

SWAT performed well during both calibration and validation periods (see Supplementary Materials and Figure S1). WetQual performance for ${\text{NO}}_{3}^{{}^{-}}$ was also at an acceptable level at the two sites where observed data were available (Section S6 of Supplementary Materials).

**Figure 4.**
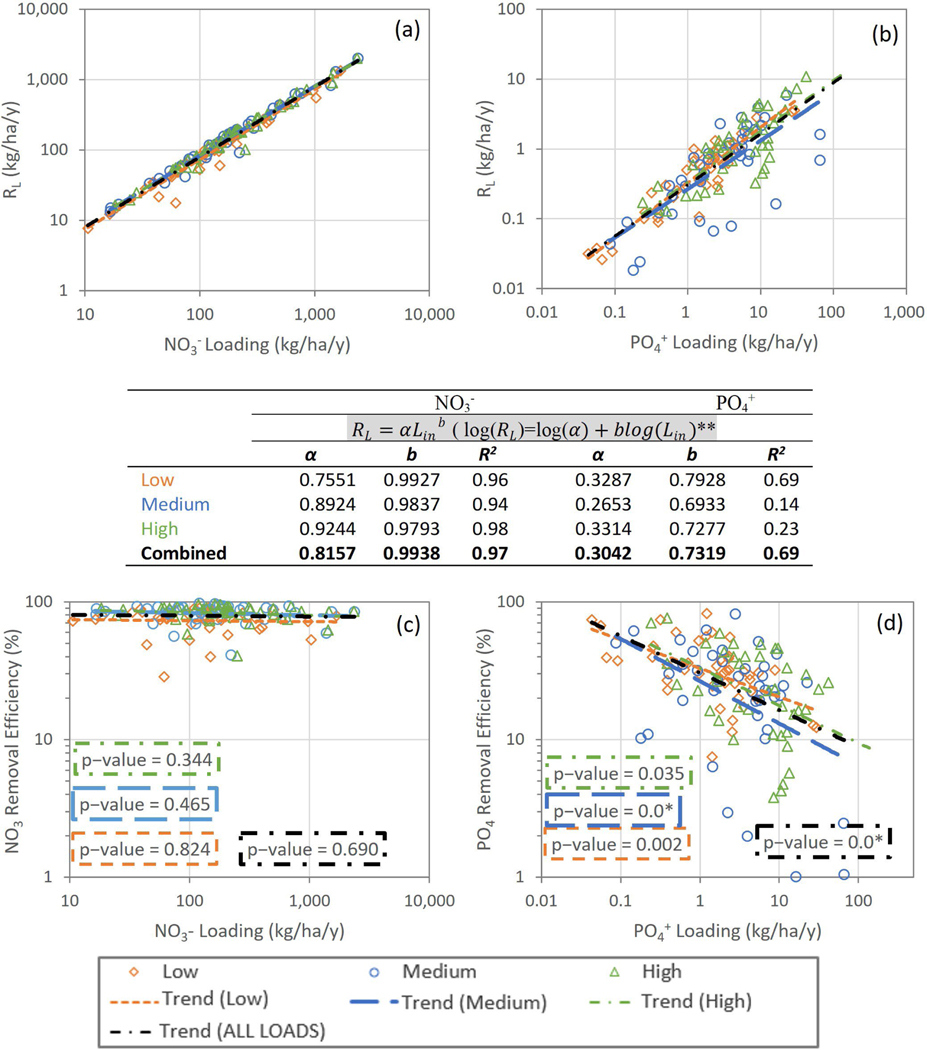
Wetland ${\text{NO}}_{3}^{{}^{-}}$ and ${\text{PO}}_{4}^{+}$ (**a**,**b**) load reduction $\left(R_{L}\right)$ vs. input loads (kg/ha/y) and (**c**,**d**) removal efficiency $\left(E_{R}\right)$ vs. input loads (kg/ha/y). The scatter was obtained from the median of WetQual-simulated average annual loads for low, medium, and high SWAT-CUP-simulated loading timeseries. $R_{L}$ is the nutrient load reduction, and $L_{in}$ is the nutrient load into a wetland. *: a *p*–value of “0.0” denotes a computed value less than 0.001. **: all slopes are significant at α=5% level.

Figure 4a,b shows calculated annual nutrient removal $R_{L}\left(\text{kg}/\text{ha}/\text{y}\right)$ versus the average annual nutrient input loads (kg/ha/y) $\left(L_{in}\right)$ of ${\text{NO}}_{3}^{{}^{-}}$ and ${\text{PO}}_{4}^{+}$ under low, medium, and high loading conditions for the period of 2008–2015. The equations of the trend lines were constructed by taking logarithms with base 10.$R^{2},\alpha ,b$, and *p*–values of the slope of the trend lines are given in Figure 4. $R_{L}$ and $L_{in}$ were highly correlated for ${\text{NO}}_{3}^{{}^{-}}$ under all loading conditions. The $R^{2}$ values of the trend lines ranged from 0.94 to 0.98. ${\text{NO}}_{3}^{{}^{-}}$ removals under all loadings showed a statistically significant increasing trend with load (*p <* 0.05). The coefficient *α* of $R_{L}$ under high loadings was greater than that under low and medium loadings. Since the power (=slope in log-transformed form) (*b*) values of the regression lines were almost equal to 1.0 (varied between 0.979 and 0.994), it can be concluded that the 44 wetlands in the UFRW yield higher ${\text{NO}}_{3}^{{}^{-}}$ removals under high loadings, which is not surprising. The slopes of the trend lines were also compared to one another. We found no difference between the slopes (all *p*–values > 0.05) under different loadings. The relationship for ${\text{NO}}_{3}^{{}^{-}}$ removals considering low, medium, and high input loadings combined was $R_{L}=0.8157L_{\mathrm{in}}^{0.9938}$ (single equation for all 44*3 = 152 points).

Figure 4b presents ${\text{PO}}_{4}^{+}\;R_{L}$ versus $L_{in}$ relationships under low, medium, and high input loadings. The $R^{2}$ values of the trend lines varied, with low loading having a much stronger relationship than medium and high loading cases. ${\text{PO}}_{4}^{+}\;R_{L}$ was positively correlated (*p* < 0.05) with $L_{in}$ under all loading conditions. The coefficient α of removals under medium loadings was smaller than that for medium and high loadings, and a similar pattern was observed for the slopes (Figure 4). However, there was no difference among them (*p* > 0.05). ${\text{PO}}_{4}^{+}$ load removals were estimated after aggregating the input loadings as $R_{L}=\;0.3042\;L_{\mathrm{in}}^{0.7319}$ .

As shown in Figure 4c,d, $E_{R}$ versus the corresponding nutrient input loads $\left(L_{in}\right)$ were plotted to assess the variation of removal percentages under low, medium, and high loadings of ${\text{NO}}_{3}^{{}^{-}}$ and ${\text{PO}}_{4}^{+}$. ${\text{NO}}_{3}^{{}^{-}}\;E_{R}$ calculated for the 44 wetlands varied between 29–91%, 40–97%, and 41–96%, with averages (medians) and ± standard deviation of 75 (78)% (±14%), 84 (87)% (±11%), and 84 (86)% (±10%) under low, medium, and high loadings, respectively (Figure 4c). The $E_{R}$ for ${\text{NO}}_{3}^{{}^{-}}$ did not show a relationship with the input loads (*p >* 0.05). Figure 4d shows the relationship between ${\text{PO}}_{4}^{+}\;E_{R}$ and $L_{in}$. $E_{R}$ varied between 7–82%, 1–82%, and 4–76%, with averages (medians) of 35 (32)% (±18%), 27 (25)% (±18%), and 28 (23)% (±18%) under low, medium, and high loadings, respectively. Two wetlands under medium loadings and three wetlands under high loadings, which were not reported in the graphs, did not show any ${\text{PO}}_{4}^{+}$ removal, respectively. ${\text{PO}}_{4}^{+}\;E_{R}$ decreased with increasing phosphorus inputs under all loading conditions (*p* < 0.05).

### 4.2. Nutrient Removal Efficiencies and Wetland Characteristics

**Figure 5.**
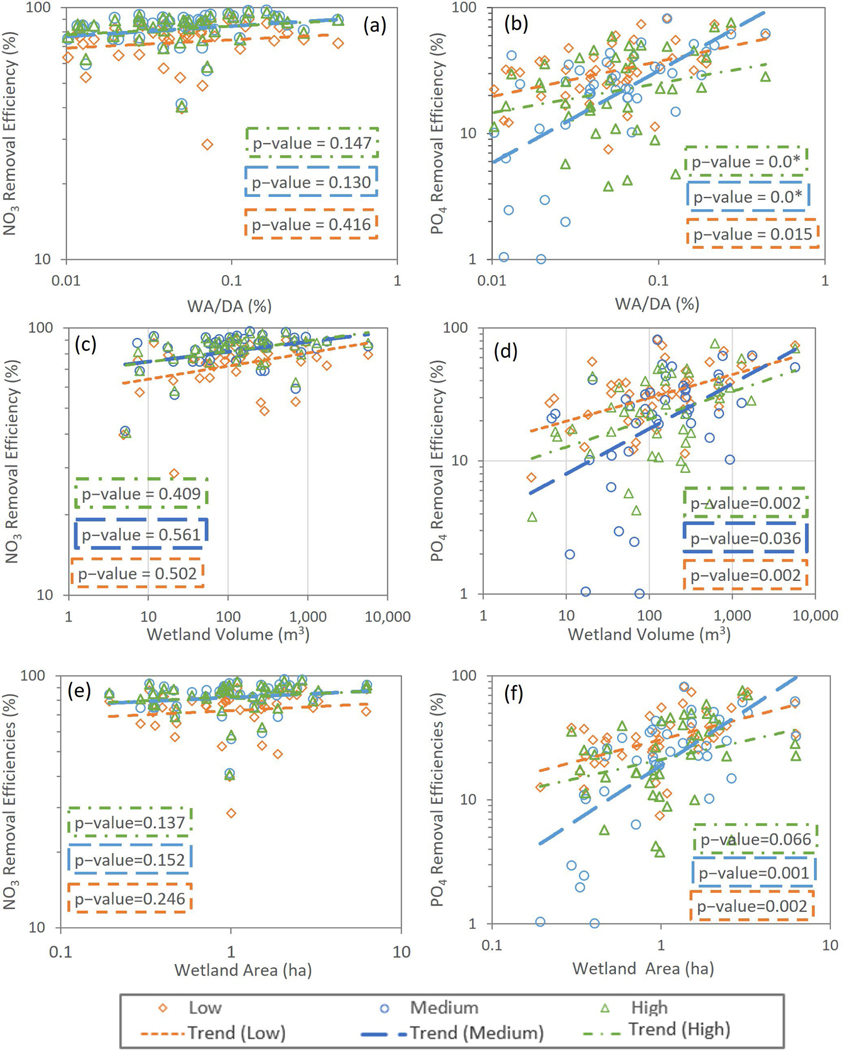
Nutrient removal efficiencies versus (**a**,**b**) wetland area/drainage area, (**c**,**d**) wetland volume (m^3^), and (**e**,**f**) wetland area (ha). The scatter was obtained from the median of WetQual-simulated annual average loads for low, medium, and high SWAT-CUP-simulated loading timeseries. *: a *p*-value of “0.0” denotes a computed value less than 0.001; WA: wetland maximum area; DA: wetland drainage area.

**Figure 6.**
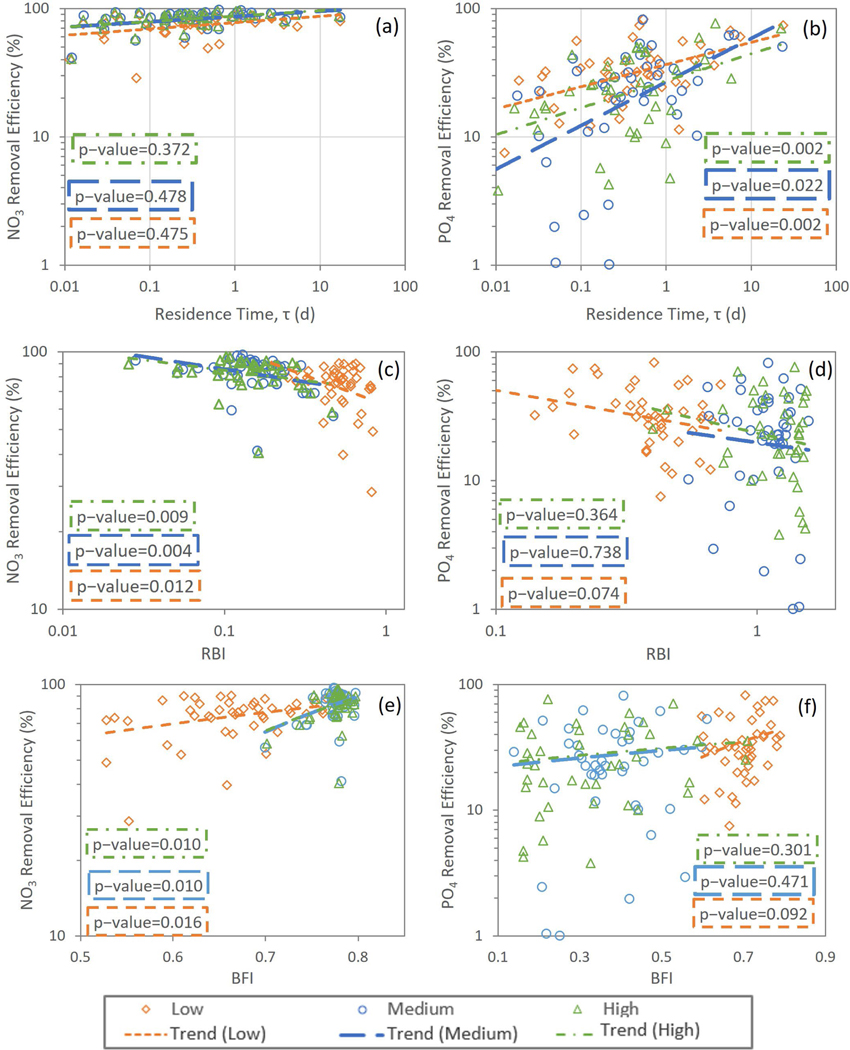
Nutrient removal efficiencies versus (**a**,**b**) residence time (day), (**c**,**d**) Richards–Baker index (RBI), and (**e**,**f**) baseflow index (BFI). The scatter was obtained from the median of WetQual-simulated average annual loads for low, medium, and high SWAT-CUP-simulated loading timeseries.

Figures 5 and 6 show *E*_*R*_ versus wetland physical and hydraulic characteristics, respectively, for low, medium, and high loadings into the UFRW wetlands. Figure 5a,b shows the relationships between nutrient $E_{R}$ and ratios of wetland area to contributing drainage area (WA/DA). ${\text{NO}}_{3}^{{}^{-}}\;E_{R}$ did not have a significant relationship with WA/DA under any of the three loadings scenarios (low, medium, and high) (Figure 5a). ${\text{PO}}_{4}^{+}\;E_{R}$ increased significantly with WA/DA under all three loading conditions (Figure 5b). Figure 5c,d shows the relationships between $E_{R}$ and wetland volumes for ${\text{NO}}_{3}^{{}^{-}}$ and ${\text{PO}}_{4}^{+}$. Results were similar to the relationships with WA/DA, i.e., ${\text{NO}}_{3}^{{}^{-}}\;E_{R}$ was not impacted by the wetland volume (*p* > 0.05 for all loadings), whereas ${\text{PO}}_{4}^{+}\;E_{R}$ increased with wetland volumes regardless of the loading (*p* < 0.05 for all scenarios). Figure 5e,f shows the relationships between nutrients $E_{R}$ and wetland areas. The relationships for both nutrients and the differences between them were the same as those reported for the other physical characteristics shown in Figure 5a–d.

The relationships between nutrients $E_{R}$ and residence time (τ) are shown in Figure 6a,b. For ${\text{NO}}_{3}^{{}^{-}}$, there were no significant relationships with τ for any of the loading scenarios, but the lowest loading did show a slightly more apparent positive slope. On the other hand, ${\text{PO}}_{4}^{+}\;E_{R}$ increased significantly with τ(p<0.05) under all loading scenarios. Figure 6c,d shows the relationships between nutrient $E_{R}$ and RBI. ${\text{NO}}_{3}^{{}^{-}}\;E_{R}$ decreased significantly with RBI (*p* < 0.05) for all loading scenarios, while the relationships between ${\text{PO}}_{4}^{+}\;E_{R}$ and RBI were insignificant for all of the loading scenarios (*p* > 0.05). Similarly, for BFI, relationships with ${\text{NO}}_{3}^{{}^{-}}\;E_{R}$ were significant for all scenarios but were not for ${\text{PO}}_{4}^{+}\;E_{R}$. However, for the ${\text{NO}}_{3}^{{}^{-}}\;E_{R}$, and in contrast to the RBI, the relationship with BFI was positive (*p* < 0.05) (Figure 6f).

### 4.3. Impact of Dry/Wet Years

**Figure 7.**
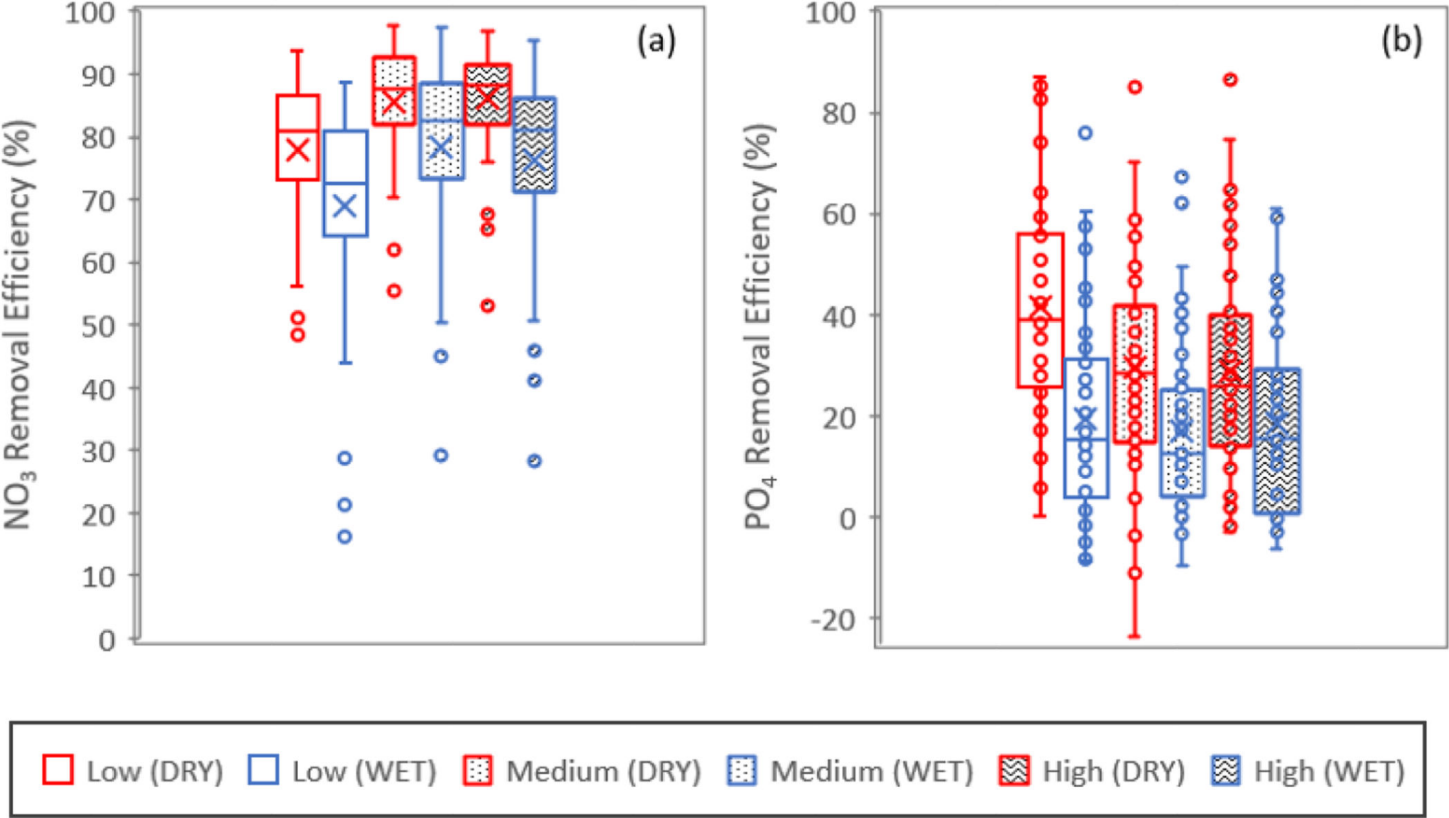
Comparisons of removal efficiencies in dry and wet years under low, medium, and high loadings for (**a**) nitrate and (**b**) phosphate. The removal efficiency was calculated from the median of WetQual-simulated average annual loads for low, medium, and high SWAT-CUP-simulated loading timeseries. In the legend, the first word indicates the loading (low, medium, or high), and the word in parenthesis indicates whether it was a wet or dry year. For example, “Medium (WET)” refers to the box plot created using removal efficiencies from all 44 wetlands under medium load during the wet year (2014).

We compared $E_{R}$ in a dry (2011) and wet (2014) year to determine whether drier/wetter conditions impacted the wetland nutrient removals under the three different loading scenarios (Figure 7). In this case, we plotted $E_{R}$ as box and whisker distributions using the removal efficiency values calculated for the 44 wetlands modeled. There were significant differences between the medians of the $E_{R}$ distributions for both ${\text{NO}}_{3}^{{}^{-}}$ and ${\text{PO}}_{4}^{+}$ in dry and wet years (*p* < 0.05) and under all loading scenarios. The magnitude of the differences in $E_{R}$ between dry and wet years was much greater for ${\text{PO}}_{4}^{+}$ compared to for ${\text{NO}}_{3}^{{}^{-}}$ (Figure 7a vs. Figure 7b), which reflects the broader range of $E_{R}$ for ${\text{PO}}_{4}^{+}$ compared to that for ${\text{NO}}_{3}^{{}^{-}}$. The ${\text{PO}}_{4}^{+}\;E_{R}$ also had some negative values (more during the wet year), which indicates that wetlands can act as a source for ${\text{PO}}_{4}^{+}$ during high flows. Overall, all the wetlands had a higher $E_{R}$ for ${\text{NO}}_{3}^{{}^{-}}$ and ${\text{PO}}_{4}^{+}$ in the dry year compared to in the wet year.

## 5. Discussion

Since the mid-20th century, the Southeastern U.S. has witnessed accelerated urbanization rates, usually outpacing average urban growth in the conterminous U.S. [61]. The UFRW has experienced an approximate 90% increase in urban area since 1992 according to NLCD data. The population in coastal Alabama increased by 65% from 2000 to 2020, and this trend is expected to continue (www.census.gov, accessed on 1 February 2022). In this context, it is reasonable to assume that the UFRW is primed to experience water quality deterioration, which could lead to the impairment of water resources and the loss of wetland ecosystems. As a result, it is vital to understand the importance of green infrastructures such as natural wetlands in water quality protection. The Weeks Bay Watershed Management Plan (WBWMP) [41] outlines a comprehensive approach to address the issues and concerns identified for the land and water areas within the Weeks Bay watershed. One of the recommendations is implementing nutrient load reduction management measures through BMPs and wetland restoration with expanded SWAT data analysis. This study helps to inform decisions related to nutrient management implementation by investigating the effects of headwater wetlands on nutrient reductions in the UFRW through the integration of SWAT and WetQual models.

**Figure 8.**
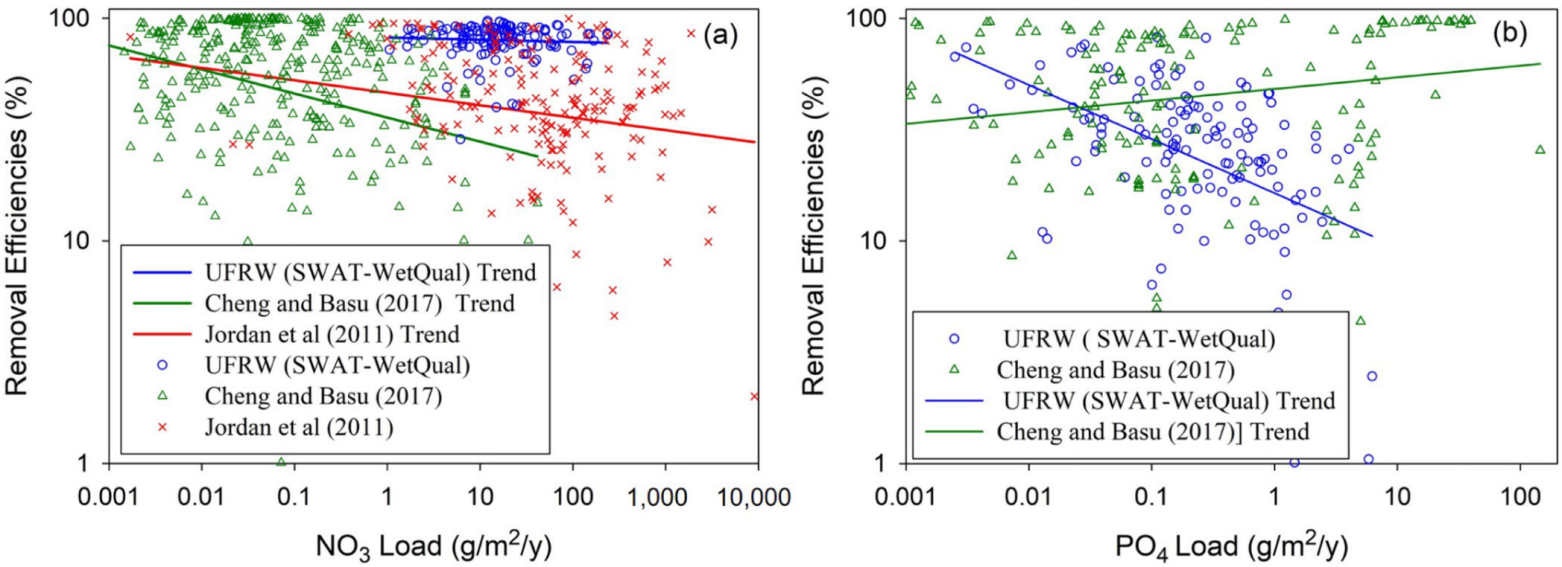
(**a**) Comparison of ${\text{NO}}_{3}^{{}^{-}}$ removal efficiencies in this study with those in Jordan et al. [11] and Cheng and Basu [9]. (**b**) Comparison of ${\text{PO}}_{4}^{+}$ removal efficiencies in this study with those in Cheng and Basu [9]. UFRW: Upper Fish River watershed. One marker is used for nitrate removal efficiencies in the UFRW wetlands for all low, medium, and high loadings.

While the stream, lake, and reservoir water resource management communities have converged on recognizing the significantly greater role of smaller systems in global nutrient processing, there has been relatively less research exploring the role of system size on nutrient processing for wetlands [9]. Our study explored the variation of nutrient removal efficiencies with input loads and the impact of wetland characteristics on nutrient retention with uncertainty consideration in the UFRW. The computed medians of ${\text{NO}}_{3}^{{}^{-}}$ and ${\text{PO}}_{4}^{+}$ load reductions obtained by the MC method for each loading scenario factor in uncertainty due to modeling errors and the sparse data used to calibrate SWAT and WetQual for the UFRW. The nutrient removal efficiencies we calculated using a quasi-probabilistic approach for the UFRW wetlands under low, medium, and high loadings are consistent with those values reported in Jordan et al. [11] and Cheng and Basu [9], as shown in Figure 8.

Jordan et al. [11] and Cheng and Basu [9] carried out exhaustive meta-analyses on wetlands to explore correlations of nutrient removal rates with nutrient loading and dependence of nutrient removal rates on system sizes, respectively. Jordan et al. [11] collected reactive nitrogen removal data from 190 wetland sites around the world. Cheng and Basu [9] compiled nitrate, total nitrogen, phosphate, and total phosphorous removal data from 600 sites, including wetlands, lakes, and reservoirs, around the world. We compared our nitrate and phosphate removal results with their findings. To accomplish this, we excluded all lakes, reservoirs, and wetlands with negative removal rates.

${\text{NO}}_{3}^{{}^{-}}$ wetland $E_{R}$, calculated by the SWAT-WetQual model framework in the UFRW, varied between 29% and 97%, with an average of 81% (±12%) under all loading scenarios. Although on average higher, the SWAT-WetQual-calculated median values were within the range of values provided in the observational-based studies by Jordan et al. [11] and Cheng and Basu [9]. The two studies, respectively, reported a ${\text{NO}}_{3}^{{}^{-}}\;E_{R}$ ranging from 0.2 to 99.6% and from 1 to 99.9%, with averages of 46.7% (±25%) and 61% (±30%), respectively (Figure 8a). The significantly larger average $E_{R}$ calculated for the UFRW indicates that our values tended to be larger compared to the average and range of values reported by Jordan et al. [11] and Cheng and Basu [9]. The $E_{R}$ standard deviation for the UFRW wetlands was much smaller, indicating less uncertainty associated with it compared to the two global studies, which should not be a surprise because our study focused on one HUC12 watershed with the same physiography throughout, while the others were more global and included several wetland types around the world. Nonetheless, ${\text{NO}}_{3}^{{}^{-}}\;E_{R}$ in the UFRW tended to decrease with ${\text{NO}}_{3}^{{}^{-}}$ loading, which is consistent with the findings by Jordan et al. [11] and Cheng and Basu [9], even though this trend in our model-based study was not significant (*p* > 0.05). The loadings per unit area considered by Cheng and Basu [9] were much smaller than our model-based estimates, whereas Jordan et al. [11] reported much higher loads (Figure 8a).

We also compared the SWAT-WetQual-based phosphate removal efficiencies with those of Cheng and Basu [9], as illustrated in Figure 8b. Model-computed ${\text{PO}}_{4}^{+}\;E_{R}$ for the UFRW varied between 1% and 82%, with an average of 30% (±18%), while in Cheng and Basu [9], they ranged from 0.5 to 99.8%, with an average of 58% (±32%). Contrary to the results for nitrate, the ${\text{PO}}_{4}^{+}\;E_{R}$ values calculated for the UFRW were skewed lower than those reported by Cheng and Basu [9]. However, if we only consider the natural wetlands in Cheng and Basu [9], where they reported 26.3% (±19.2%) ${\text{PO}}_{4}^{+}$ removal, the results are very similar. The standard deviation of ${\text{PO}}_{4}^{+}\;E_{R}$ that we predicted for the UFRW is significantly smaller than that reported by Cheng and Basu [9]. ${\text{PO}}_{4}^{+}\;E_{R}$ in both the UFRW and Cheng and Basu [9] had significant relationships with ${\text{PO}}_{4}^{+}$ loading (*p* < 0.05) but in opposite directions, decreasing and increasing with loadings, respectively. The cluster of high removal efficiencies and high loadings likely influenced the direction of the slope in the regression for the Cheng and Basu [9] data (Figure 8b). If those were removed, the slope would have turned downward and been more consistent with the URFW results.

The results obtained from this modeling study suggest that smaller wetlands are as efficient as larger wetlands in removing ${\text{NO}}_{3}^{{}^{-}}$ loads (Figure 5e). Considering the abundance of small wetlands, this is a significant finding. In contrast to the ${\text{NO}}_{3}^{{}^{-}}$ results, wetland size matters a great deal to ${\text{PO}}_{4}^{+}\;E_{R}$ (Figure 5f). Similarly, ${\text{NO}}_{3}^{{}^{-}}\;E_{R}$ has no relationship with the ratio of wetland area to wetland drainage area (WA/DA) or volumes, whereas the ${\text{PO}}_{4}^{+}\;E_{R}$ has a significant increasing trend with WA/DA and volumes under all loadings. As highlighted by Ramesh et al. [18], smaller wetlands may be more vulnerable to environmental changes, and thus special attention should be given to these ecosystems, especially in coastal areas such as southern AL, where urbanization trends may negatively impact water quality in the future.

We also explored variations in nutrient removal efficiencies in a dry and wet year under low, medium, and high loadings. Although the model-calculated nutrient removal efficiencies showed some variations with both ${\text{NO}}_{3}^{{}^{-}}$ and ${\text{PO}}_{4}^{+}$ and for different loading levels, calculated removal efficiencies were consistently higher in the dry year in all wetlands. The dry year also was on average 1.1 °C warmer than the wet year. The WetQual model allows higher denitrification rates with increased temperatures using the Arrhenius equation [28]. WetQual simulates higher plant growth rates under higher temperatures, which leads to an increased level of N uptake by plants. Lower precipitation and higher evapotranspiration values can also increase nutrient concentrations in wetlands, which leads to higher estimated N uptake by plants in WetQual. In WetQual, N uptake is proportional to the inorganic N concentration in the water. Therefore, if the concentration is low, the plants uptake less N, and if the concentration is high, plants uptake more N.

Studies in the literature reported seasonal effects on nutrient removal efficiencies. For instance, Windolf et al. [62] found that nitrogen removals were generally higher during summer in 16 shallow Danish lakes. Similarly, Spieles and Mitsch [63] found that nitrate removal efficiencies were most efficient during summer in three wetlands in central Ohio, USA. Kalin et al. [29] demonstrated interannual nutrient reductions for a 2-year period in a restored wetland located on Kent Island, Maryland. They estimated that both ${\text{NO}}_{3}^{{}^{-}}$ and ${\text{PO}}_{4}^{+}$ load removals in a drier year were higher than those in the wetter year. Ulén et al. [64] also reported higher phosphorus and nitrogen removal efficiencies in summer in constructed wetlands in Stockholm, Sweden. It is worth highlighting that the summer months in the aforementioned studies were dryer than the winter months, which indicates that the seasonal variations in nutrient removal efficiencies are consistent with the results presented in the current study.

Our model-based results are in line with those reported in previous studies [9,11,12] and can be transferred to similar wetland systems. Since studies quantifying nutrient loads and removal efficiencies in natural wetlands are limited [65], our findings have provided new insights into nutrient removal in headwater wetlands and offer tools for predicting nitrate and phosphate removal in similar wetlands located within sparsely gauged or ungauged watersheds.

We also used the regressed power relationships in Figure 4a,b to extrapolate nutrient removals to the relatively smaller 348 non-riverine/riparian wetlands within UFRW that were not modelled using the SWAT-WetQual model framework. We realize that such extrapolations were made in the company of a high degree of uncertainty. Forty-one percent of the UFRW drains to these wetlands. As a first step in extrapolating nutrient reduction, we developed a multi-regression relationship between SWAT nutrient loads at the outlets of 1529 sub-basins and their land use/cover (LULC) in the UFRW under low, medium, and high loading conditions. Next, we calculated loadings to the 348 wetlands in the UFRW using their LULC distributions as input to the developed regression relationships. Then, we estimated $R_{L}$ by applying the equations given in the table under Figure 4a,b. Table 1 summarizes the estimated total nutrient loadings to these wetlands and the total removed loads under low, medium, and high loading conditions. Results showed that these wetlands collectively are estimated to remove 51–61% of the ${\text{NO}}_{3}^{{}^{-}}$ and 5–10% of the ${\text{PO}}_{4}^{+}$ loading they receive from their respective drainage areas.

**Figure 9.**
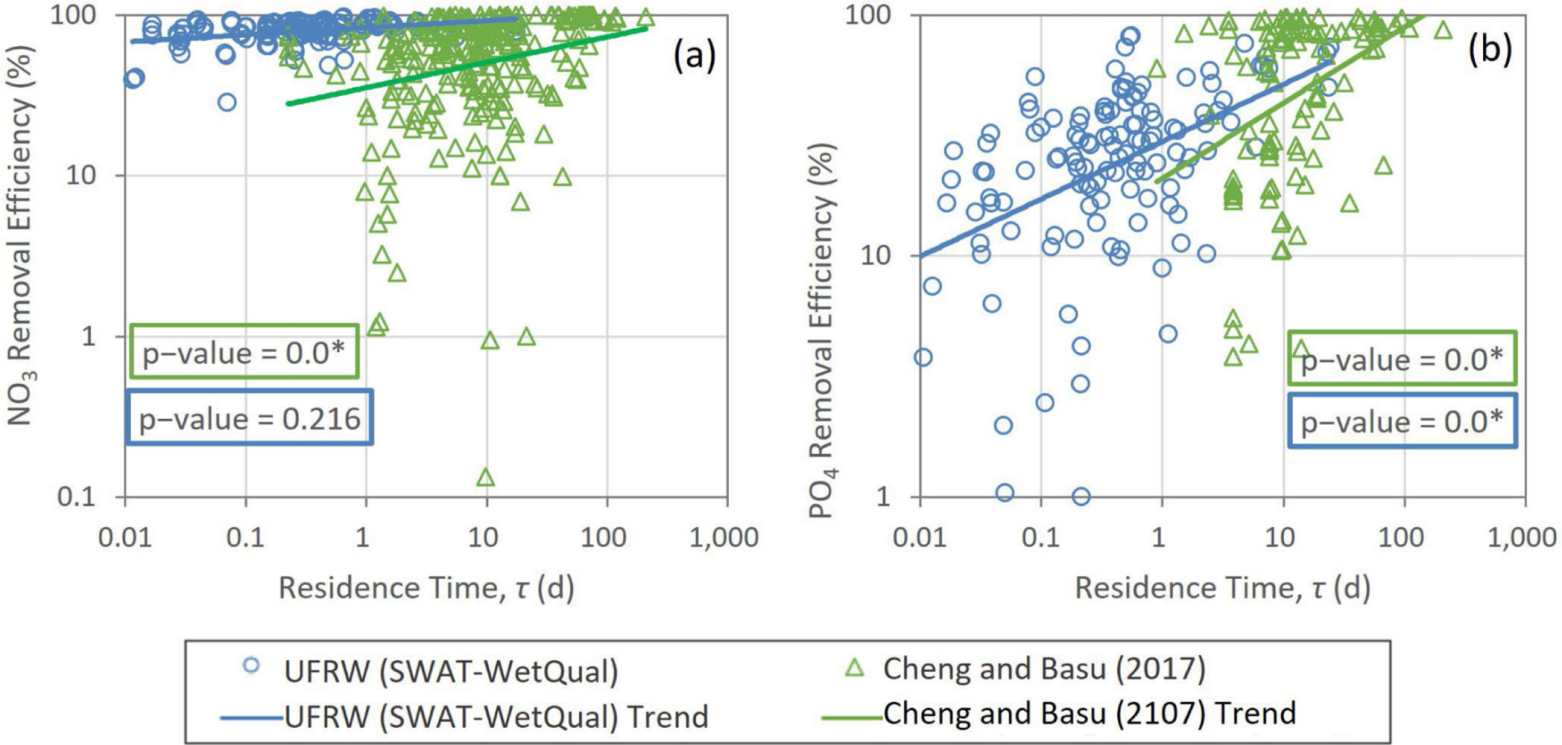
Comparison of removal efficiency versus residence time in this study and those in Cheng and Basu [9] for (**a**) ${\text{NO}}_{3}^{{}^{-}}$ and (**b**) ${\text{PO}}_{4}^{+}$. UFRW: Upper Fish River watershed. One marker is used for nitrate removal efficiencies in the UFRW wetlands for all low, medium, and high loadings. *: a *p*–value of “0.0” denotes a computed value less than 0.001.

Finally, we compared removal efficiencies versus residence time in this study and those in Cheng and Basu [9], who reported the residence time of their wetlands. ${\text{NO}}_{3}^{{}^{-}}$ removal efficiencies in the UFRW did not increase with residence time, while those in Cheng and Basu [9] did (Figure 9a). However, the slopes of the trend lines between the two studies were statistically not different (*p* > 0.05). ${\text{PO}}_{4}^{+}$ removal efficiencies showed a statistically significant but different increasing trend (*p* < 0.05) with residence time in both UFRW and Cheng and Basu [9] (Figure 9b). However, the data reported by Cheng and Basu [9] for ${\text{NO}}_{3}^{{}^{-}}$ and ${\text{PO}}_{4}^{+}$ were skewed toward wetland systems with longer residence time and showed a greater variance for nitrate removal efficiency than the estimates obtained for the UFRW. Although the majority of residence times in the UFRW wetlands were lower than those reported in Cheng and Basu [9], the slopes of the regressions with residence time for ${\text{PO}}_{4}^{+}$ were similar. This suggests that estimates of ${\text{PO}}_{4}^{+}$ removal efficiencies obtained from estimates of residence time can be considered reasonably robust for planning purposes.

The relatively shallow water table environment and the subtropical climate of coastal Alabama may partly explain the higher nitrate removal efficiency values calculated for the UFRW compared to the global nitrate removal rates reported by Cheng and Basu [9]. The high temperature, abundant organic matter contents, and persistent anaerobic conditions all act to push nitrate removal rates higher. The calculated residence time for most of the wetlands in the UFRW ranged between 0.1 and 1 day (the majority closer to 1 and higher), and some were as high as 10 days, as shown in Figures 6a and 9a. Considering the empirical formula reported by Cheng and Basu [9] for first-order nitrate removal rate, $k_{{\mathrm{NO}}_{3}^{-}}=0.63\;\tau^{-0.86},k_{{\mathrm{NO}}_{3}^{-}}=0.63-4.56/\text{day}$ for τ ranging between 1 and 0.1 days and as small as 0.087/day for τ=10 days. The nitrate removal rates calculated by the above formula [0.087/day–0.63/day] likely accounted for denitrification and other loss pathways and, hence, are consistent with the overall smaller values of 0.004–0.15/day calibrated for the first-order denitrification rate in the two study wetlands (Supplementary Materials, Table S3). In essence, our (model-based) denitrification rates are on the lower side of the overall nitrate removal rates produced by the global formula.

These comparisons show that despite the sparsely gauged and monitored UFRW and the localized nature of this model-based study, the results predicted by the SWAT-WetQual-MC framework in general are consistent with the observed global data and hence complement the literature on wetland nutrient function.

## 6. Summary and Conclusions

Nutrient loads, nutrient load removals, and nutrient removal efficiencies of 44 headwater wetlands were investigated in this study under low, medium, and high loading conditions. This was achieved by implementing a watershed–wetland model framework, MC simulations, and statistical regression to estimate nutrient reduction in a sparsely monitored watershed. The following summarizes the key findings:

Our model-based results demonstrate that the headwater wetlands in the study region may, on average, have removal efficiencies of 81% (±12%) and 30% (±18%) for ${\text{NO}}_{3}^{{}^{-}}$ and ${\text{PO}}_{4}^{+}$, respectively.

Computed mean ${\text{NO}}_{3}^{{}^{-}}$ removals were highly correlated with their input loads with an almost linear relationship of $R_{L}=0.8157L_{\mathrm{in}}^{0.99}$ for aggregated low, medium, and high loads, but removal efficiencies showed no significant trend with their input loads under any of the loading scenarios. This was likely a function of the predicted high removal efficiencies across the board. For ${\text{PO}}_{4}^{+}$, load removals were positively correlated with their input loadings for all loading scenarios with a power relationship of $R_{L}=0.3042\;L_{\mathrm{in}}^{0.73}$, while ${\text{PO}}_{4}^{+}$ removal efficiencies showed a significant decreasing trend with input loadings under low, medium, and high loadings.

Considering the shallow water table environment and subtropical climate in coastal Alabama, the relatively higher denitrification rates obtained in this study are not at odds with the total nitrate removal rates calculated by the empirical formula obtained by Cheng and Basu [9] based on more global wetland nitrogen data.

By developing a multivariate regression model relating nutrient loads to LULC/land use in the UFRW, the statistical *R*_*L*_ relationships were used to extrapolate ${\text{NO}}_{3}^{{}^{-}}$ and ${\text{PO}}_{4}^{+}$ removal efficiencies to 348 riverine–riparian wetlands in the watershed that were not modeled by the SWAT-WetQual framework. Estimated removal efficiencies of 51–61% for ${\text{NO}}_{3}^{{}^{-}}$ and 5–10% for ${\text{PO}}_{4}^{+}$ indicated that smaller headwater wetlands are as effective as larger wetlands in removing nutrients.

Calculated ${\text{NO}}_{3}^{{}^{-}}$ removal efficiencies did not show a significant trend with wetland area/drainage area, wetland volume, and residence times under all loading conditions, whereas they did show a significant trend with RBI and BFI. Calculated ${\text{PO}}_{4}^{+}$ removal efficiencies were positively correlated with wetland area/drainage area, wetland volume, and residence time under all loadings (*p* < 0.05), while they showed an insignificant trend with RBI and BFI under all loading conditions.

The 44 study wetlands modeled yielded higher removal efficiencies for both ${\text{NO}}_{3}^{{}^{-}}$ and ${\text{PO}}_{4}^{+}$ in the dry year compared to the wet year simulations under all loading scenarios.

Although these model-based findings are localized to the UFRW, they, in general, are consistent with those by Cheng and Basu [9] and Jordan (2011), which were based on more global datasets. The greater spread and variance in their global data may explain the differences to the localized results obtained for the UFRW.

By virtue of geographical proximity and physiographic similarity, the regression models developed for the UFRW and the findings in this study can be scaled up to the coastal plains of Alabama and Northwest Florida. The current study offers new insights to the modeling community into the importance of natural wetlands as pollution sinks in coastal regions.

## Data Availability Statement:

Publicly available datasets were analyzed in this study. The source of the data can be found in Table S1 and in papers [9,11].

## Supplementary Material

Supplementary Material

## Figures and Tables

**Table 1.** Estimated ${\text{NO}}_{3}^{{}^{-}}$ and ${\text{PO}}_{4}^{+}$ loadings and removals for the 348 wetlands in the UFRW under low, medium, and high conditions. These wetlands were not used in integrated SWAT-WetQual modeling. Load removal and removal efficiency were calculated using the regression equations in Figure 4 developed with calculated data from the 44 selected wetlands.

	${\text{NO}}_{3}^{{}^{-}}$ (kg/yr)			${\text{PO}}_{4}^{+}$ (kg/yr)		

	Low	Medium	High	Low	Medium	High

*Load in*	45,523	61,586	68,973	645	1457	2650
*Removal*	23,403	37,375	41,903	62	67	134
$E_{R}\left(\%\right)$	51%	61%	61%	10%	5%	5%

$E_{R}$: removal efficiency; UFRW: Upper Fish River watershed.
