# Supplementary material for: Nutrient Removal Potential of Headwater Wetlands in Coastal Plains of Alabama, USA
Source: Water (Basel). Author manuscript; Available in PMC 2024 Jul 25. (PMC10569132; doi:10.3390/w15152687)
Supplement: Supplementary Material [file NIHMS1925153-supplement-Supplementary_Material.pdf]

SUPPLEMENTARY MATERIAL

S1. SWAT model

SWAT is a process-based, semi-distributed watershed-scale eco-hydrological model developed by the United States Department of Agriculture (USDA) Agricultural Research Service (ARS[27-52]. Major model input components include weather, hydrology, soil properties, plant growth, water quality, and land management [66]. SWAT was originally developed for the prediction of the long-term impact of rural and agricultural management practices on water, sediment, and agricultural chemical yields in large, complex watersheds with varying soils, land use, and management conditions. As a semi-distributed model, SWAT divides the watershed into sub-watersheds, which are further discretized into hydrologic response units (HRUs). The HRUs represent the smallest spatial unit in SWAT and correspond to a unique combination of soil, LULC, and slope. A detailed description of the processes simulated by SWAT can be found in the SWAT theoretical manual [52].

S2. SWAT model setup

ArcSWAT Version2012.10.4.21 (https://swat.tamu.edu, accessed on April 1 2020) was used to set up the Upper Fish River Watershed (UFRW) model. The data used in the model setup and calibration are summarized in *Table S1*. Geospatial data with a 10-meter resolution Digital Elevation Model (DEM), soil data, and the 2016 National Land Cover Database were used in the SWAT model. National Hydrography Dataset (NHD) was used to delineate watersheds in the SWAT model. Based on downloaded crop layers from USDA National Agricultural Statistics Service (https://nassgeodata.gmu.edu/CropScape, accessed on April 15 2020) we observed that the most common crop rotation trend in the region is peanut-cotton and cotton-peanut. Therefore, the management file of the SWAT model was set up with this configuration in agricultural areas. Management operations including fertilizer applications and their timing were based on Butler and

Srivastava [67] for South Alabama. Grazing operations were added in pasture and range HRU's and livestock was assumed to be only beef cattle. Annual manure quantities were calculated based on livestock population estimated by the National Agricultural Statistics Service [68]. The SWAT model includes 1,529 sub-basins, 44 wetlands, and 15,529 HRUs. Daily streamflow data was downloaded from the United States Geological Survey (USGS) water data website for the period 2004-2015 and was used in calibrating SWAT.

Since approximately 35% of the UFRW area is covered by Evergreen, Deciduous, and Mixed Forest we made modifications in the SWAT plant database to improve forest modeling. Our forest parameterization was based on recent efforts by Yang and Zhang [69], Yang et al. [70], and Haas et al [71]. We adjusted several parameters related to Leaf Area Index (LAI), ET, and biomass.

Atmospheric deposition data were downloaded from the National Atmospheric Deposition Program (NADP) website (http://nadp.slh.wisc.edu, accessed on April 20 2020) for the sites FL96 (Pensacola, Florida), AL02 (Baldwin, Alabama), and AL24 (Mobile, Alabama). Irregular data was available from 2001 to 2016 and averaged values for the period 2008-2015 were used as input data. Instream Nitrate (NO_3^-) and PO_4^+ data were downloaded from the National Water Quality website (https://www.waterqualitydata.us) for the USGS station at the UFRW outlet. Since they were not regularly available for the simulation period, we transformed the available instantaneous NO_3^- and PO_4^+ concentrations into continuous monthly loadings using the USGS's Load Estimator (LOADEST) software [72], which is a widely used program for estimating constituent loads in streams and rivers. Given a time series of streamflow, additional data variables, and constituent concentration, LOADEST assists the user in developing a regression model for the estimation of the constituent load. The outputs generated by LOADEST were then used for monthly sediment and NO_3^- calibration for the period 2008-2015.

S3. WetQual model

WetQual is a process-based model which simulates hydrologic processes as well as N, P, total suspended sediment (TSS), and C cycles and their dynamics in natural and constructed wetlands [28-30,73,26]. The model partitions a wetland into three basic compartments: (1) water column, (2) wetland soil layer (which is further portioned into aerobic and anaerobic zones), and (3) plant biomass. WetQual simulates oxygen dynamics and the impact of oxidizing and reducing conditions on nitrogen transformation and removal, and approximate phosphorus precipitation and releases into soluble forms under aerobic and anaerobic conditions, respectively. Additionally, the model accounts for volatilization and denitrification as nitrogen loss mechanisms. Plant biomass growth and decay are simulated based on a simple mass balance equation and free-floating biomass is separated from rooted aquatic plants. The daily growth rate is computed based on a simple model for productivity in which the daily growth rate is related to daily solar radiation. The model runs on a daily time scale, while the model internally divides the one-day time interval into a smaller time interval for numerical integration. A detailed description of WetQual can be found in Hantush et al. [28], Kalin et al. [29], and Sharifi et al. [26,30]. WetQual requires hydro-climate data and input concentrations which were fed by SWAT model outputs such as inflow, precipitation, evapotranspiration, water temperature, and nutrient concentrations. A FORTRAN script was written to automate the multiple processes of reading, computing, and writing from SWAT model outputs to WetQual inputs. The WetQual model parameters were borrowed from Kalin et al [29] and they were calibrated using observed data from 2 nearby headwater wetlands which were explained under the “WetQual Calibration” section below.

The studied wetlands have mainly pine-forested uplands. Bischoff et al [74] estimated the dry weight biomasses in wetlands as 2 tons/ha for foliage (leaf) and 24 tons/ha for wood. They also estimated the nitrogen productions for foliage and wood at 70 (0.035% of foliage biomass) and 60

(0.0025% of woody biomass) kg/ha/y, respectively. They also gave foliage biomass of 3.5 tons/ha/y from uplands with 80 kg/ha/y nitrogen production. Clawson et al [75] gave total litterfall biomasses of 3.5-9 tons/ha/y for wetlands in the Southeastern United States. Accordingly, we assumed that 10% and 90% of biomasses are from foliage and wood, respectively, with initial biomass of 16 tons/ha in the forested wetlands. We also assumed that gross primary productivity (GPP) is 6 tons/ha/y biomass for foliage and wood in wetlands with a 3 tons/ha/y net primary productivity (NPP) based on the suggested values in the literature and personal communications (Dr. Graeme Lockaby, wetland biochemist at Auburn University).

S4. Model Assessments

The performances of the SWAT and WetQual models were evaluated with the Nash–Sutcliffe efficiency (*NSE*) and percent bias ratio (*PBIAS*):

$$NSE = 1 - \frac{\sum(O_i - S_i)^2}{\sum(O_i - \bar{O}_i)^2} \qquad PBIAS = \frac{\sum(O_i - S_i)}{\sum(O_i)}$$

Where, O and S represent observed and simulated variables of interest, and $\bar{O}$ the mean of observed data. For more information regarding the employed statistical rating metrics, the reader is referred to Moriasi et al. [76] and Moriasi et al. [77].

S5. SWAT calibration and validation

SWAT-CUP was used to stochastically calibrate SWAT for flow, NO_3^- and PO_4^+. For each, multiple iterations of 500 simulations were performed until improvement in model performance (*NSE*) was negligible. The final iterations led to the calibrated model parameter ranges. The SWAT model was run from 2004 to 2015, with the first 4 years being used as a warm-up period. Thus, the model's performances were calculated considering the simulation period 2008-2015. *Figure S1* shows the modeled and observed monthly streamflow, NO_3^-, and PO_4^+ from 2008 to 2015. The monthly flow,

NO_3^-, and PO_4^+ were stochastically calibrated and validated with SWAT-CUP using observed data at the watershed outlet between 2008-2011 and 2012-2015, respectively. The model performances for the best simulations are shown in *Table S2*. *Figure S1a* shows the modeled and observed monthly streamflow for calibration and validation periods. *NSE* and *PBIAS* for streamflow were 0.86 and 2%, respectively, for the calibration period while they were 0.95 and 4%, respectively, for the validation period. These statistics fall within the very good performance rating suggested by Moriasi et al. [77]. The model's calibration of NO_3^- and PO_4^+ was carried out by adjusting key water quality-related parameters commonly reported in the literature [48,78-82]. Observed and simulated NO_3^- and PO_4^+ are shown in *Figure S1b* and *S1c* for calibration and validation periods. The model parameters for NO_3^- and PO_4^+ were optimized to maximize the model performance (NSE). The model's skills in predicting NO_3^- and PO_4^+ can be classified as very good [77], considering the achieved *NSE* values (Table S2) while NO_3^- and PO_4^+ performances can be considered as good and very good, respectively, considering the achieved *PBIAS* values.

S6. WetQual calibration

WetQual model parameters were stochastically calibrated between 2013-2014 for nitrate load at two headwater wetlands, which are very close to the UFRW, in Baldwin County, Alabama. No phosphorus data were available at these sites; therefore, calibration was not performed for phosphorous parameters. Ramesh et al [18] described hydrologic and nitrate data collection in Old Foley wetland (OF) located at the headwaters of Graham Creek, and Bay Minette wetland (BM). We ran an MC simulation (10,000 model runs) for each wetland using the default parameter ranges and distributions under the WetQual graphical user interface (GUI) (https://github.com/USEPA/WetQual-GUI) [83]. The WetQual-GUI produces the 20-most sensitive parameters and their dotty plots which show model performance versus the range of the sensitive parameters [83], which are useful to get new parameter ranges for the most

sensitive parameters. The ranges of the first 9 parameters listed in *Table S3* were initially updated using the dotty plots of NO_3^-. Based on the revised ranges, another MC simulation (10,000) was run and parameter ranges were updated based on the best 10% model simulations. Model performance measures used to determine the best 10% model simulations was the *NSE*. The last 3 parameters in *Table S3* were modified based on forest information and dry-weight biomass values in the study wetlands. *Table S4* shows best model performances for NO_3^- at the two wetlands. WetQual had much better model performance at BM than NF. Since BM is also the closest wetland to the UFRW, we used the final parameter ranges for BM.

S7. Assessment of wetlands removal rates

Table S5 provides a summary of nutrient input/output loads, and removals at wetlands under low, medium, and high loadings which were described under the “Methods” section in the main text. “Low”, “Medium”, and “High” columns are input loads to wetlands and other columns are output loads (and removal loads) from wetlands.

References

References are given in the Main text.

Tables

Table S1. SWAT input data

Input data	Source	description
**Topography**	USDA Geospatial Data gateway https://datagateway.nrcs.usda.gov/	10 meters resolution DEM
**Soil**	USDA NRCS https://websoilsurvey.sc.egov.usda.gov	County-level Soil Survey Geographic Dataset (SSURGO)
**Hydrography**	National Hydrography Dataset (NHD) https://gdg.sc.egov.usda.gov	National Hydrography Dataset (NHD) v2. Source: USDA Geospatial Data Gateway
**Wetland Data**	National Wetland Inventory (NWI) https://www.fws.gov/wetlands/data/Mapper.html	National Wetland Inventory (NWI) wetland data mapper
**Cropland Data**	USDA CropScape https://nassgeodata.gmu.edu/CropScape/	Cropland Data Layer (2011 CDL)
**Climate**	PRISM Climate Group, Oregon State University https://prism.oregonstate.edu	Daily temperature and rainfall data
**Point sources**	Alabama Department of Environmental Management (ADEM) http://www.adem.state.al.us	Loxley wastewater treatment plant and Spanish Fort wastewater treatment plant water quality data
**Atmospheric deposition**	National Atmospheric Deposition Program (NADP) http://nadp.slh.wisc.edu	Wet and dry deposition of NO_3^- and NH_4 were obtained from NADP
**Streamflow**	USGS water data https://waterdata.usgs.gov/nwis	Daily streamflow data

Table S2. SWAT model performances for monthly streamflow, nitrate, and phosphate load calibrations.

		**Streamflow**	$\mathbf{NO_3^-}$	$\mathbf{PO_4^+}$
Calibration (2008-2011)	*NSE*	0.86	0.28	0.89
	PBIAS (%)	2	6	-2
Validation (2012-2018)	*NSE*	0.95	0.83	0.81
	PBIAS (%)	4	-1	-14

Table S3. The final ranges of calibrated parameters for the WetQual model.

**Symbol in publications**	**Definition, Units**	**Min**	**Max**
k_{gb}	Growth rate of a benthic and rooted plant (1/day)	0.0009	0.005
k_{mr}	First-order rapid mineralization rate in wetland soil (1/day)	0.000001	0.0002
k_{nw}	First-order nitrification rate in wetland-free water (1/day)	0.0001	0.3
k_{mw}	First-order mineralization rate in wetland-free water (1/day)	0.000001	0.0001
k_{dn}	Denitrification rate in anaerobic soil layer (1/day)	0.004	0.15
v_{ss}	Effective settling velocity (cm/day) for sediment	2.6335	2633.5
f_{act}	Vertical diffusion magnification factor	9.3	5000
α_{ro}	Coefficient for resuspension/recycling of organic material	0.00001	10
α_{rs}	Coefficient for resuspension/recycling of sediment	0.00001	0.05
l_2	Thickness of anaerobic soil layer (cm)	5	100
a_{na}	Gram of nitrogen per gram of chlorophyll-a in plant/algae (gN/gChl)	0.005	0.01
a_{pa}	Ratio of phosphorus to Chlorophyll-a in algae (grP/grChl)	0.0005	0.001

Table S4. WetQual calibration performances for NO_3^- loads at the two wetlands in Baldwin County, Alabama.

**Wetlands**	***NSE***	***PBIAS* (%)**
BM	0.94	2
OF	0.11	-6

BM: Bay Minette wetland; OF: Old Foley wetland

Table S5. Summary statistics for nutrient input and output loads at 44 wetlands. Values within paranthesis indicate load removals (kg/ha/y).

		Loadings to Wetlands	Output (Removal) Loads			Loadings to Wetlands	Output (Removal) Loads			Loadings to Wetlands	Output (Removal) Loads		
		**Low**	**L-10%**	**L-50%**	**L-90%**	**Medium**	**M-10%**	**M-50%**	**M-90%**	**High**	**H-10%**	**H-50%**	**H-90%**
		**kg/ha/year**											
NO_3^-	min	10.6	2.3 (8.3)	3 (7.6)	3 (7.6)	16.6	1.6 (13.1)	1.8 (13.5)	1.8 (11.9)	18.3	1.7 (16.7)	1.9 (16.4)	2.1 (16.2)
	max	1684	492 (1329)	484 (1333)	454 (1337)	2375	502 (2055)	562 (2003)	623 (1996)	2348	650 (1994)	536 (2008)	668 (1951)
	ave	222	57 (164)	60 (162)	62 (160)	301	50 (251)	56 (245)	61 (240)	335.5	61 (275)	62 (274)	67 (269)
	stdev	314	97 (230)	95 (231)	91 (236)	450	92 (376)	104 (366)	113 (364)	443.4	113 (358)	99 (363)	119 (353)
PO_4^+	min	0.04	0.01 (0.02)	0.01 (0.03)	0.01 (-0.02)	0.09	0.01 (-0.24)	0.04 (-1)	0.05 (-4.2)	0.24	0.07 (-1.6)	0.07 (-7)	0.10 (-16.2)
	max	29.4	26 (7.1)	25.8 (3.6)	27.3 (2.1)	65.8	58 (11.1)	65 (5.9)	70 (3.5)	138.5	118 (22.5)	142 (11)	155 (4.5)
	ave	3.2	2.2 (1.0)	2.5 (0.7)	2.6 (0.5)	7.3	5.5 (1.8)	6.3 (1)	6.9 (0.4)	14.1	10.9 (3.2)	12.6 (1.5)	14.2 (-0.1)
	stdev	5.8	4.7 (1.3)	5.1 (0.8)	5.4 (0.5)	13.6	11.7 (2.5)	13.4 (1.2)	14.1 (1.2)	26.3	22.9 (4.8)	27.2 (2.6)	29.3 (3.5)

The table presents minimum, maximum, average, and standard deviation values of input, output, and removal loads at the 44 wetlands.

Low, Medium, and High are nutrient loads at wetland inlets at the 10th, 50th, and 90th percentile of SWAT-CUP simulations, respectively.

X-10%, X-50%, and X-90% are output loads (and removal loads) at the 10th, 50th, and 90th percentiles of WetQual simulations at wetland outlets under X loadings. X is L, M, or H for Low, Medium, and High loadings, respectively.

Figures

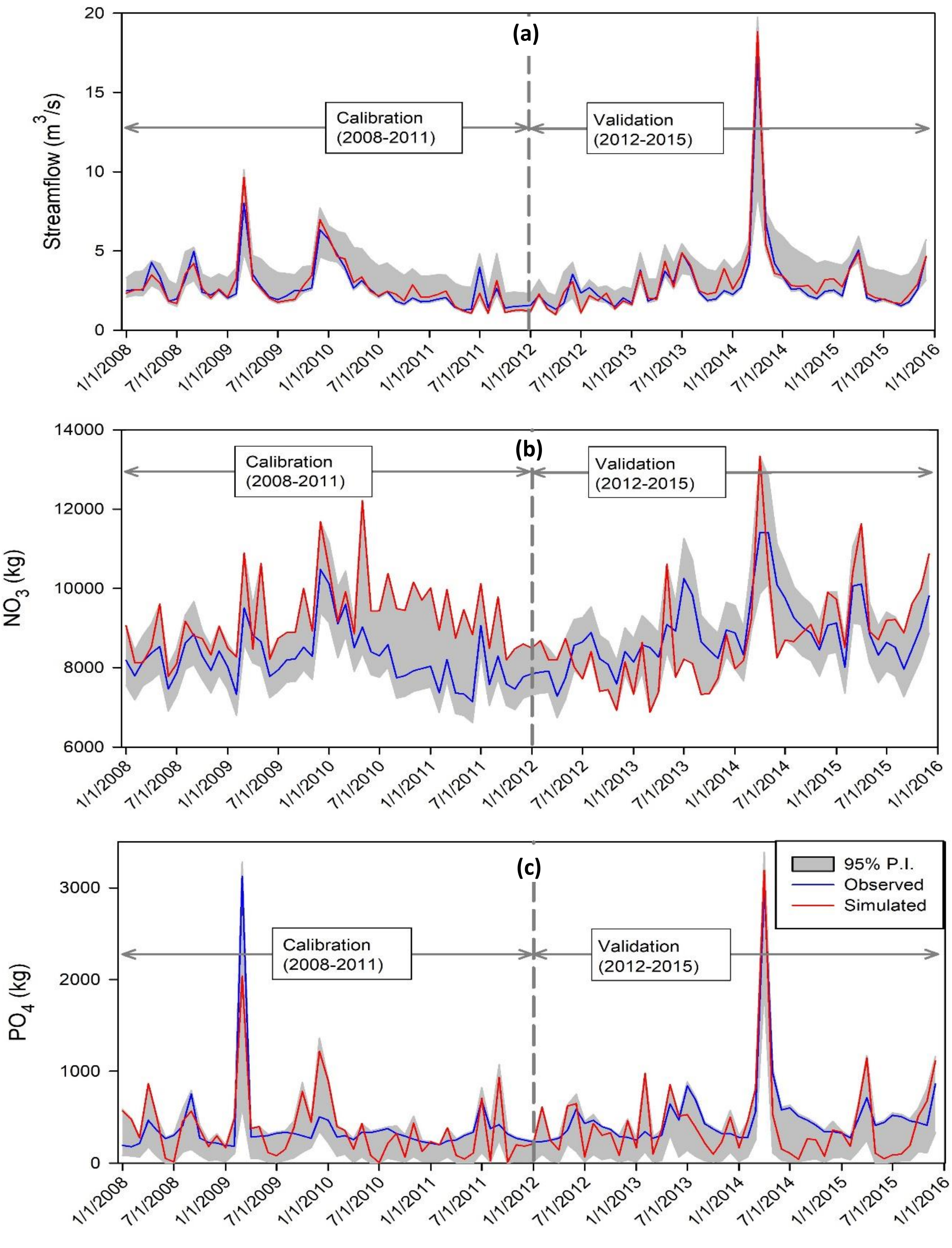

Figure S1. Simulated and observed monthly (a) streamflow, (b) NO_3^-, and (c) PO_4^+ loads at the UFRW outlet.
